# Comparing the efficacy of probiotic plus antibiotic with the Antibiotic Therapy alone on the recurrence of Bacterial Vaginosis

**DOI:** 10.12669/pjms.41.1.9922

**Published:** 2025-01

**Authors:** Saima Ashraf, Ammara Haroon, Asiya Fayyaz

**Affiliations:** 1 Saima Ashraf Associate Professor, Department of Obstetrics and Gynecology, Nishtar Hospital, Multan, Pakistan; 2 Ammara Haroon Department of Obstetrics and Gynecology, Nishtar Hospital, Multan, Pakistan; 3 Asiya Fayyaz Department of Obstetrics and Gynecology, Nishtar Hospital, Multan, Pakistan

**Keywords:** Antibiotics, Bacterial Vaginosis, Metronidazole, Probiotics, Recurrence, Recurrent Vaginitis

## Abstract

**Objective:**

To compare the efficacy of a probiotic plus antibiotic with antibiotic therapy alone for the prevention of the recurrence of bacterial vaginosis.

**Method:**

A Randomized control trial was conducted in the Gynecological Department of Nishtar Hospital, Multan, between July 2022 and June 2023. One hundred and twenty (N=120) women with bacterial vaginosis, all cured with metronidazole, were enrolled and randomized. In Group-A, Probiotics were administered once a day for 14 days following antibiotic treatment (Tab Metronidazole 400mg, three times a day for seven days). In Group-B, only oral antibiotics were given. The patients were followed up in four visits for five months for bacterial vaginosis recurrence (responders, partial responders, non-responders, and drop-outs) using Amsel criteria by vaginal swabs. All the data was collected on a structured Performa.

**Results:**

The mean age of enrolled 120 women was 32.28±2.58, and 111(92.5%) were married. Of 120 enrolled women, 8 (6.7%) were dropped out. Evaluating the recurrence rate, the probiotic group (n=57) had fewer recurrences than the placebo (n=55) (8.9% vs 21.4%, p-value <0.05). Group-A had a better responder rate at five months of follow-up than the placebo group (39.2% vs. 25.8%) (RR 0.31, 95%CI: 0.1450-0.7998, *p*-Value <0.05). Comparing the effect of probiotics on the components of the Amsel criteria, only vaginal discharge had a statistically significant reduction by using probiotics plus antibiotics (*p*-value≤0.05).

**Conclusion:**

This trial showed that probiotics plus antibiotics significantly reduced bacterial vaginosis recurrences than antibiotics alone.

## INTRODUCTION

Bacterial Vaginosis (BV) accounts for 30% of vaginal discharge in childbearing women.^1^ It is caused by an imbalance of vaginal micro-ecology and Gardnerella vaginalis (GV) and anaerobic bacteria from irrigation, several sexual partners, non-condom usage, smoking, and low estrogen levels.^2^,^3^ BV symptoms include increased vaginal discharge, fishy leucorrhea smell, irritation, and vulva burning. Some studies have connected BV to premature birth, pelvic inflammation, infection, and STIs, including AIDS. Because BV causes vulva discomfort and recurs frequently, it adversely damages women’s life quality and mental health.^4^ About half of BV patients have clinical symptoms that the Amsel criteria or Nugent score may identify. Amsel is a straightforward and practical approach utilized as the gold standard in clinics.^5^

BV patients’ vaginal microbiota has changed as *Lactobacillus* has evolved into a more varied population of facultative and obligate anaerobic bacteria. Clinically, metronidazole and clindamycin are used worldwide to kill BV-related bacteria and restore normal vaginal microbiota swiftly.^6^ After effective antimicrobial treatment, 69% recurred. Gut troubles, nausea, vomiting, and antibiotic resistance might result.^7^ Safety and longevity in BV treatment must be investigated. Probiotics are a safe and well-tolerated option to restore the micro-ecological balance of the female reproductive tract. Even though additional randomized controlled trials employing probiotics as an alternative or complementary therapy for BV are described, their effectiveness is debatable. Past research has also shown disparities in probiotic dose.^7^,^8^

**Fig.1:**
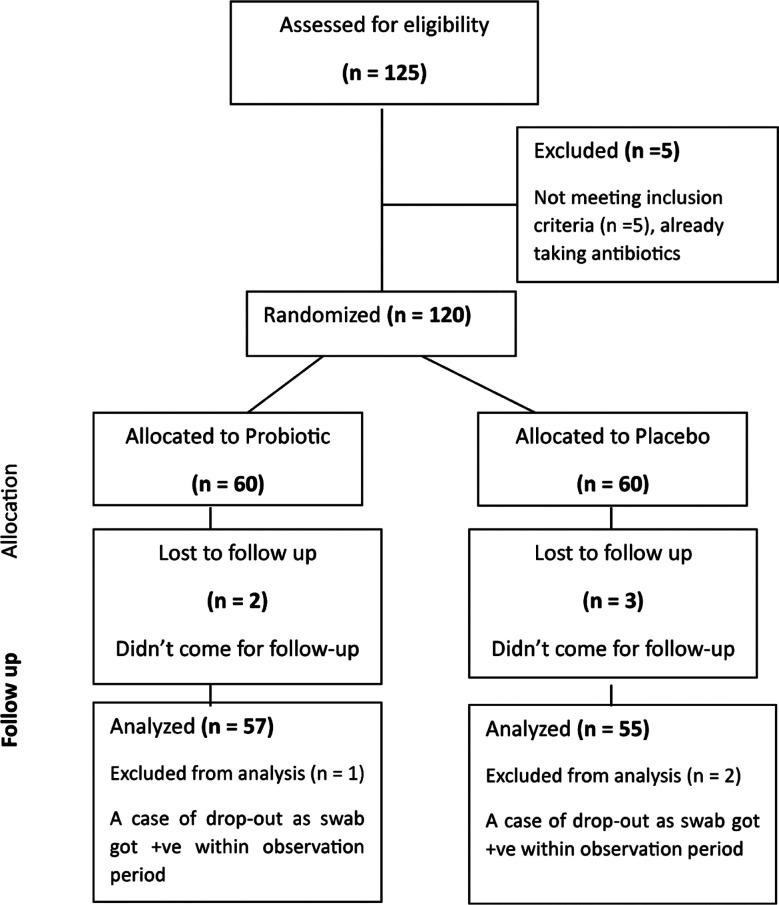
Consort Layout of the trial.

There is a need to enhance the knowledge of our population to meet international standard criteria, using more local knowledge and up-to-date international data. So, this study aimed to compare the efficacy of probiotics plus antibiotics with antibiotic therapy alone to prevent the recurrence of bacterial vaginosis.

## METHOD

A randomized control trial was conducted in the Gynecological Department of Nishtar Hospital, Multan, between July 2022 and June 2023, by using a simple random sampling technique. The sample size was calculated using the recurrence rate of BV treatment with probiotics to antibiotics (16% vs 40%=104, 52 in each group).^9^ Women between 18-45 years of age having BV diagnosed according to Amsel’s criteria (at least three of the following BV symptoms: Greyish white vaginal discharge, positive amine test findings, vaginal pH >4.5, and presence of clue cells (>20%) in a wet smear) and all cured with metronidazole were enrolled. Patients with a history of allergy against the study drug**,** pregnant, lactating, menopausal women**,** history of bleeding from the genital tract of unknown etiology**,** women with malignancy of reproductive tract or any co-morbid condition like AIDS, diabetes, psychiatric illness or currently using any contraceptive method patients taking another oral/vaginal probiotic or getting antibiotics were excluded.

### Trial Registration & EC Approval:

The study was approved by Institutional Ethical Review Board clearance (Number 095; Dated 01/03/2022) and trial registration (IRCT20230814059147N1, Iranian Registry of Clinical Trial, Iran).

After history and examination, 120 patients were enrolled to undergo vaginal swabs for pH, wet mount, and sniff tests. A color change was seen on litmus paper when measuring vaginal pH and clue cells on wet mounting. The sniff test was passed by adding 10% potassium hydroxide (KOH) solutions to the discharge on a clean glass slide and observing for a fishy solid scent. The Amsel Criteria was used to diagnose BV. A lottery separated enrolled subjects into two groups. In Group-A, Probiotics (Lactobacillus plantarum PBS067, Lactobacillus rhamnosus LRH020, and Bifidobacterium animalis lactis BL050) were administered once a day for 14 days following antibiotic treatment (Tab Metronidazole 400mg TDS for seven days). A post-antibiotic therapy (same antibiotic in same dose and duration) and a placebo (Dextrose-filled pill) were administered to Group-B (n=52). It was a single-blinded study, where patients were given drugs in colored envelopes, i.e., green for Group-A and red for Group-B. Each of the four follow-ups included BV vaginal swabs. First, enroll BV-diagnosed women for metronidazole therapy; 2) Verify antibiotic effectiveness (Amsel <3) and allocate probiotic or placebo groups. Reintegration: one capsule/day for 14 days.

### Monitoring and maintenance:

One capsule is taken each day for seven days a month after menstruation for four months. Third, Amsel-defined BV recurrence was assessed at baseline and final visits to determine whether the participant had BV difficulties after the study or throughout the treatments. Recurrence was defined as BV after at least one menstrual cycle (28 days) from clinical verification of healed BV after an episodic regimen. Recurrence rate split patients: Responder = five months without BV relapses on antibiotics. Partial responders had one incidence throughout the four-month observation period, while non-responders had many. Subject withdrawal due to personal or compliance issues; relapse during reintegration (the first 14 days of probiotics). On organized Proforma, data was collected.

Data was analyzed using SPSS v26. For qualitative variables (like treatment response, recurrence, and any drop-out), percentages were calculated. Mean and standard deviation were used for quantitative variables like age. The normality of the data was checked by using the Shapiro-Wilk test. Quantitative variables between the two were analyzed using an independent sample t-test. For the qualitative variable, the Chi-square test was used. Relative risk reduction was also calculated, incorporating partial as a positive outcome and excluding dropouts. In the end, the association of each component of Amsel criteria was compared between the two groups using the Chi-square test, and a P-Value ≤0.05 was considered significant.

## RESULTS

The mean age of enrolled 120 patients was 32.28±2.58, while range of age was 28-38 years. Based on division into two groups, the probiotics group patients were slightly older than the placebo group (33.75±2.38 vs 30.85±1.92). There were 111(92.5%) married patients, while only 9(7.5%) were unmarried.

Out of 120 enrolled patients, 8(6.7%) dropped out, three got positive swab tests within the observation period, and five abandoned the trial. So, 57 patients in the probiotics and 55 in the placebo group were available for evaluation in the trial. Evaluating the recurrence rate, the probiotic group had fewer recurrences than the placebo (8.9% vs. 21.4%). A comparison of both groups in terms of mean age, marital status, and recurrence rate is given in Table-I.

**Table-I:** Comparison of Both Groups.

Variable		Probiotic Group (n=57)	Placebo Group (n=55)	Test value	p-Value
Mean Age		33.65±2.4	30.92±1.9	2.900*	0.00
Marital status	Married	57 (50.9%)	0 (0%)	8.929**	0.003
	Unmarried	47 (42%)	8 (7.1%)
Recurrence (partial+ Non-Responders)	Yes	10 (8.9%)	24 (21.4%)	9.014**	0.003
	No	47 (42%)	31 (27.7%)

* = t-test;

** = chi-square.

Comparing the probiotics’ efficacy with antibiotics in the BV recurrence, the former group had a better responder rate at five months follow-up (39.2% vs. 25.8%), a statistically significant association. Probiotics have a relative risk of not curing BV of 0.31 compared to antibiotics. (Relative Risk (RR): 0.34 (95%CI=0.1450 to 0.7998; *p-Value* = 0.02) (Table-II)

**Table-II:** Comparison of the Outcome Between Two Groups.

	Study Group		Total

	Probiotic (n=60)	Placebo (n=60)	
Responder	47 (39.2%)	31 (25.8%)	78 (65%)
Partial Responder	4 (3.3%)	7 (5.8%)	11 (9.2%)
Non-Responder	6 (5%)	17 (14.2%)	23 (19.2%)
Drop out	3 (2.5%)	5 (4.2)	8 (6.6%)
Total	60 (50%)	60 (50%)	120 (100%)

RR Antibiotic to Probiotic: 0.31 (95%CI= 0.1450 to 0.7998; Chi-Square value:9.328; p-value = 0.02).

Comparing the effect of probiotics on the components of the Amsel criteria, in this case, probiotics had a more significant reduction in clue cell positivity, more negative pH results, lesser discharges, and more negative amine test results but only vaginal discharge had statistically significant reduction by using probiotics (*p*-value<0.05). (Table-III)

**Table-III:** Comparison of Both Groups in Terms of each component of AMSEL Criteria.

AMSEL’s Criteria		Study Groups		Total (n=122)	Chi-Square value	p-Value

		Probiotics (n=57)	Placebo (n=55)			
Clue Cell Positivity	Yes	9 (15.8%)	15 (27.3%)	24 (21.4%)	2.192	0.139
	No	48 (84.2%)	40 (72.7%)	88 (78.6%)		
Vaginal Discharge	Yes	7 (12.3%)	18 (32.7%)	25 (22.3%)	3.442	0.016
	No	50 (87.7%)	37 (67.3%)	87 (77.7%)		
Positive P^H^ Criteria	Yes	10 (17.5%)	18 (32.7%)	28 (25%)	5.769	0.064
	No	47 (82.5%)	37 (67.3%)	84 (75%)		
Positive Amine Test	Yes	10 (17.5%)	16 (29.1%)	26 (23.2%)	2.094	0.148
	No	47 (82.5%)	39 (70.9%)	86 (76.8%)		

## DISCUSSION

A woman’s vaginal ecosystem is invaded from birth until death. Reproductive-aged women generate 1-4 mL of vaginal fluid with 108–109 bacterial cells.^10^ Vaginal discharge, itching, and malodor affect 50% of BV patients.^11^ BV has been diagnosed using Nugent, Hey-Ison, and a computer model that detects and classifies gram stains using Nugent criteria.^12^ Despite having poorer sensitivity and specificity than molecular genetics, Amsel criteria are still the most often utilized in clinical settings because they can be done rapidly at the bedside.^1^

Sexual contact is a risk factor for bacterial vaginosis, according to various research.^13^ According to our study, the BV was diagnosed in women who were married (90%). However, post-pubertal women who have never been sexually active develop BV at a lower rate than those who do (77% vs 33%).^14^ Research found a strong association between age and BV, with age ≤20 (7 (95% CI, 1.57-31.79)) and age 31-35 (0.5 (95% CI, 0.30, 0.92).^15^ Another study reported about 71.57% of female patients under the age of 19 had the highest incidence of G. vaginalis.^16^

According to our study, metronidazole alone treated BV at 52%, but adding probiotics enhanced effectiveness to 78%, a statistically significant difference. One research found that metronidazole reduced *G. Vaginalis* incidence by 20% by reducing vaginal inflammation and promoting normal lactobacillus microbiota.^16^ Other studies have found cure rates of 58-92% after one month of therapy for antimicrobials, including metronidazole. However, our community has increasing antimicrobial resistance, which reduces efficacy.^17^ These outcomes are transitory, with recurrence or re-infection rates reaching 50% within 6-12 months of therapy. Compared to antibiotics, probiotics have a relative risk of not curing BV of 0.31 in our study. Consistent with our trial, a meta-analysis showed an RR of 1.23 compared to antibiotics alone for not curing BV. [RR=1.12, 95 percent CI (0.60, 2.07), P=0.72].^18^ Comparing the doses of probiotics, high-dose probiotics have an even more enhanced cure rate.^19^

Compared to antibiotics alone, probiotics significantly reduce BV, as proven by Amsel’s criteria. But its potential impact was on vaginal discharge, where it significantly reduced (88% vs 63%; *p-value* 0.016).^20^ Probiotics reduce the vaginal discharge (mean Nugent score 3.9 to 2.6, *P*=0.002) and *G. Vaginalis* counts (log_10_ 3.57 to 2.38; *P*=0.027) in a trial, mainly by restoring the normal microbiota.^21^

### Limitations:

The presented trial had certain limitations. Firstly, the sample size was small and single-center. Moreover, it didn’t compare the efficacy of oral vs. topical probiotics. Further trials are recommended in Pakistani populations, especially considering nutritional status and pregnancy.

## CONCLUSION

This trial showed that probiotics and antibiotics significantly reduced bacterial vaginosis recurrences than antibiotics alone. Moreover, the primary symptom of vaginal discharge was significantly reduced by the use of probiotics.

### Recommendations:

1) Consider probiotics as an addition to antibiotics in recurrent bacterial vaginosis 2) Probiotics can be considered the only treatment for mild bacterial vaginosis. 3) Further studies are recommended with longer follow-ups.

### Author’s Contribution:

**SA:** Conceived, designed, and did statistical analysis & editing of the manuscript and is responsible for the integrity of the research. **AH** and **AF:** Did data collection and manuscript writing. **SA** and **AF:** Did literature search, critical review and final approval of the manuscript.
